# Continued emphasis on quality and safety jeopardizes clinical medical physics careers in radiation oncology: What can be done about it?

**DOI:** 10.1002/acm2.12532

**Published:** 2019-01-24

**Authors:** Todd Pawlicki, Arno J. Mundt

**Affiliations:** ^1^ Department of Radiation Medicine and Applied Sciences University of California San Diego CA USA

This editorial arose out of a project with the AAPM Working Group on the Prevention of Errors. The goal was to publish an interview with one of the authors in the AAPM Newsletter about tips and tricks of leading quality and safety initiatives in the clinic.^1^ The Newsletter covered strategies of change management, working with a team, building a culture of safety, and leading when you're not the leader.

While anchored in quality and safety, the interview was fundamentally about leadership. The interview triggered some deeper thought about medical physics and the future clinical role of medical physicists, especially related to quality and safety. Over many discussions between the authors of this editorial, we came to the conclusion that the continued emphasis on quality and safety is likely a threat to the long‐term viability of clinical medical physicists in radiation oncology. Clearly, this viewpoint would benefit from some explanation.

Clinical medical physics^1^ consists of a number of routine responsibilities such as equipment calibration and QA, patient‐specific QA including treatment plan checks, weekly chart checks, and patient‐specific measurements (e.g., IMRT QA, diode measurements, etc.). Physicists lend assistance to radiation oncologists, dosimetrists, and therapists at the treatment machines for hypo‐fractionated cases, gating or 4D cases, and troubleshooting equipment faults. Some external beam treatment planning is still done by physicists as well as LDR and HDR brachytherapy planning. Ad‐hoc meetings with a patients or staff to discuss issues related to radiation dose, the associated risks of radiation exposure, or other concerns about their treatment is a part of a physicist's routine job function. Physicists are equally valuable at managing technical issues when things go wrong usually by figuring out what actually happened, performing necessary dose estimations, and recommending follow‐up actions. Some physicists spend a majority of their time on quality improvement, safety initiatives, and designing processes.

Of all these activities, only a few actually require the expertise of a physicist. Generally speaking, radiation detection and measurement requires physics knowledge and training. Calibrating radiation output for external beam or brachytherapy, for example, fall into the physicist‐required category. The far majority of a physicists’ day‐to‐day value comes from assisting our clinical colleagues and checking the work of others or checking equipment performance. The raison d’être of a physicist's job is ensuring high‐quality treatments and patient safety.

Over the past ten years or so, we thought that physicists can have a bigger and more meaningful impact on patient care by learning and implementing modern quality and safety approaches.^2^, ^3^ While this is still true, we have now come to realize that the landscape where physicists add value to patient care is much more tenuous. The reality is that the current emphasis on quality and safety may lead to a decrease in job security. There are several reasons for physicists to be concerned about the status quo.

The maturation of equipment design and manufacturing does not require as many routine checks and quality monitoring as in the past. It is likely that any remaining QA deemed essential will be largely automated. Rather than requiring an army of highly trained physicists for the purposes of equipment QA, only a small team will be needed with a couple of local or regional experts to address issues that are detected. This has already been observed in some radiology departments primarily in the community setting. The usefulness of physicists’ treatment plan quality checks will decrease with automated treatment planning and the radiation oncologist's final plan approval. Workflow and process problems that physicists work on can be effectively addressed by other professionals that are trained in quality and safety. Physicists must recognize that they are primarily facilitators of patient treatments but not absolutely essential.

There are radiation oncology departments that regularly treat patients without a physicist on site and little or no evidence exists to indicate that those departments are systematically providing subpar treatments. One cannot imagine a radiation oncology department without each patient being assigned to a radiation oncologist. The same is not true for physicists — we emphasize the obvious, that a chart or a treatment plan is not a patient. Any time you are helping the process rather than driving it, you are susceptible to being replaced or worse yet, marginalized.

The good news is that physicists have the potential to add value to patient care way beyond what they are currently providing. Physicists think differently than medically trained healthcare professionals such as radiation oncologists or nurses. This provides a perspective on the care of a patient that would uniquely and positively impact outcomes. Unfortunately, the physicist's unique perspective is not being utilized because most of their time is spent checking the work of others. Physicists need to establish a role where they are *required* for the treatment of every patient — every patient should “have a physicist” for their treatment just like they “have a physician”. The value that a physicist would bring to a patient's radiation treatment should be akin to the value that a medical oncologist, surgeon, and radiation oncologist bring to the patient's overall cancer care. To achieve this, physicists need to move in a fundamentally new direction. The first step is to establish an individual professional relationship with every patient.

Our group is taking steps in this direction by developing an initiative where a physicist has a consultation with the patient prior to simulation, then meets with the patient again prior to their first treatment, and then any time the patient has a technical question or concern during their course of treatment.^4^ Preliminary results show that physicists have a positive impact on patient care by reducing patient anxiety.^5^ While reducing treatment related anxiety is important and can be beneficial to outcomes, this is only the beginning. We are investigating other areas where physicists can directly impact patient care such as taking responsibility for treatment plan approval or target volume delineation. There are many other possible directions to expand this initiative.

Whatever the final landing place, the goal should be to augment the role of the radiation oncologist, allowing him or her to have time for other important activities that improve patient care and help advance the field. If radiation oncologists are going to have more impact on patient care, they need to see patients earlier, perhaps soon after diagnosis in a multi‐disciplinary clinic alongside surgeons and medical oncologists instead of seeing patients after they have already met with other specialists. Another way to raise the profile of the field is for radiation oncologists to take more leadership roles in the Cancer Center and Medical Center. This ensures that radiation oncology has a seat at the table where decisions are made and would benefit everyone including physicists and, most of all, patients. Radiation oncologists will need to free up time to work on these other activities. One way to achieve this is to share their current clinical responsibilities. We are suggesting that the most appropriate group to share with is the physicist. When physicists are truly integrated and required to treat patients in radiation oncology, only then they will be recognized differently by hospital administrators changing their perception of physicists as leaders in patient care. This will also result in greater access to extra‐departmental leadership positions for physicists within the hospital.

The intent of this new direction is not for the physicists to abandon their traditional responsibilities in the department but to retain their current role as technical experts while adding the additional work of direct patient care. Even though we are convinced this future will take hold, something has to change. Physicists need to modify their current approach to quality and safety to make time for direct patient care responsibilities. Physicists need to spend more time interpreting quality and safety data rather than acquiring it. But, it is not a sustainable strategy to simply transfer their current activities to other staff members such as physics assistants. Job functions that add minimal value need to be significantly modified or omitted all together. There are a host of activities that fall into this classification such as monthly and annual linac QA, patient‐specific IMRT and VMAT measurements, secondary MU calculation checks, weekly chart checks, and traditional treatment plan checks. The future physicist will be employed because of their cognitive reasoning skills and clinical experience, not primarily for their ability to work hard at ensuring quality and safety. Physicists will be essential because they have a specific and well‐defined clinical role that is required to determine how each patient should be treated with radiotherapy.

The future quality and safety role of a physicist should be one of leadership with an emphasis on quality management rather than directly checking equipment, charts, or any other parameters. The responsibility of managing quality will include all aspects of resource allocation as well as the design and oversight of the quality and safety program. Tolerance levels and targets for different clinical processes will need to be benchmarked and monitored. Perhaps the biggest challenge will be motivating and ensuring a diverse group of professionals implement and maintain the required expertise. In short, physicists need leadership and management skills to facilitate and sustain this transformation.

It goes without saying that a very different training and education regimen will be required. Even at this early stage, to perform effectively in a direct patient care role requires systematically different approach to developing clinical skills.^6^ Other aspects of clinical medicine will also have to be learned including disease progression, staging, and monitoring outcomes just to name a few. Medical physics residency training programs will need to be modified to ensure the necessary clinical experience is developed for this future role.

We fully realize there are a bevy of clinical challenges to what we are suggesting in addition to regulatory and financial issues. We are confident, however, that all of these can be addressed with leadership from the AAPM in collaboration with physician‐led professional societies. The future has never been brighter for radiation oncology and clinical medical physicists provided that together they embrace change and forge a new path.

## NOTE

1 In this editorial we are specifically referring to clinical medical physicists in radiation oncology (“physicists”) although the discussion may similarly apply to clinical physicists in imaging. Also, our discussion is focused on *routine* clinical work as opposed to clinically orientated research or less routine work such as shielding design.
